# Effect of extractions from *Ephedra sinica* Stapf on hyperlipidemia in mice

**DOI:** 10.3892/etm.2014.2117

**Published:** 2014-12-08

**Authors:** YANBO FAN, JINGJING LI, QIANG YIN, YISHENG ZHANG, HUIFANG XU, XINHUA SHI, CHEN LI, YAN ZHOU, CAIXIN ZHOU

**Affiliations:** 1 State Administration of Traditional Chinese Medicine of P.R.C (Level Three), Laboratory of Traditional Chinese Medicine Preparation, Wuhan Hospital of Traditional Chinese Medicine, Wuhan, Hubei 430014, P.R. China; 2 Hubei University of Chinese Medicine, Wuhan, Hubei 430065, P.R. China; 3 Humanwell Healthcare Group Co., Ltd., Wuhan, Hubei 430000, P.R. China

**Keywords:** ephedra alkaloids, ephedra polysaccharides, ephedra non-alkaloids, hyperlipidemia, acute toxicity

## Abstract

The aim of the present study was to investigate the hypolipidemic and antioxidant potential of ephedra extractions in diet-induced hyperlipidemic mice. Mice were fed a diet high in fat to establish the hyperlipidemic model. A total of 48 mice were randomly divided into six groups, which included the normal control, model control, positive control, ephedra alkaloid, ephedra polysaccharide and ephedra non-alkaloid groups. Intragastric administration of the respective treatments was provided continuously for four weeks and the body weight was recorded weekly. The total levels of cholesterol (TC), triglycerides (TG), high-density lipoprotein cholesterol (HDL-C) and malondialdehyde (MDA), and the activity levels of superoxide dismutase (SOD), alanine aminotransferase (ALT) and aspartate aminotransferase (AST) in the serum were recorded. In addition, changes in liver morphology and organ coefficients (ratio of organ to body weight) were evaluated, while the acute toxicity reactions of ephedra extractions were investigated using the modified Spearman-Karber method. Compared with the mice in the model control group, the weight, liver coefficient, serum levels of TC, TG and MDA, and activities of ALT and AST were significantly lower (P<0.05) in the mice in the ephedra non-alkaloid group. However, the level of HDL-C and the activity of SOD were markedly higher (P<0.05). Fatty degeneration of the liver in the ephedra alkaloid and non-alkaloid groups was notably improved compared with the model control group. The mean lethal dose (LD_50_) of ephedra alkaloids was 610 mg/kg, and the maximum tolerated dose of oral ephedra non-alkaloids in the mice was 367.5-fold larger than the clinical dosage in humans. In conclusion, ephedra non-alkaloids have therapeutic potential for the treatment of hyperlipidemia, since they are able to improve lipid metabolism and are relatively safe for use under the maximum tolerated dose.

## Introduction

Hyperlipidemia is defined as an abnormal lipid metabolism or the presence of elevated levels of fats in the blood, including total cholesterol (TC), triglycerides (TG), free fatty acids, high-density lipoprotein (HDL), very low-density lipoprotein (VLDL) and low-density lipoprotein (LDL). The increased levels of blood-fats change the density and flow of the blood, which may result in arteriosclerosis (1). VLDL and LDL are easily oxidized, and oxidized VLDL and LDL (OxLDL) promote the production of oxygen free radicals and reduce the mRNA expression of nitric oxide synthase (2,3), which are the causes for the accelerated adhesion of monocytes to endothelial cells (4). OxLDL is able to increase endothelial permeability, inhibit secretion and lower metabolism, leading to vascular endothelial cell apoptosis (5). The elevated levels of lipids also lead to pathological changes in the tunica intima and a disturbance in microcirculation. Diabetes and cardiovascular disease have been shown to be associated with hyperlipidemia (6). Therefore, lowering the levels of blood lipids has important significance for the prevention and treatment of diabetes and atherosclerosis.

Currently, lipid-lowering drugs, particularly 3-hydroxy-3-methylglutaryl-coenzyme A reductase inhibitors (statins), can effectively lower the levels of TC and LDL cholesterol, reducing the incidence rate of cardiovascular events and mortality (7). A consensus has been reached that statins are the first-line agents for the treatment of hypercholesterolemia (8,9). However, the adverse reactions of statins, including myopathy and rhabdomyolysis, should be considered (10,11). Furthermore, the hepatotoxicity of statins has been reported in a number of clinical cases (12). Thus, there is a requirement for the development of novel lipid-lowering drugs.

Ephedra has been used as a medicine in China for thousands of years (13). Ephedra plants, including *Ephedra sinica* Stapf, *Ephedra intermedia* schrenk et C.A. Meyer and *Ephedra equisetina* Bge, may be used to treat colds, hay fever, allergies, pneumonia, asthma and bronchitis (14–16). Since ephedra or ephedrine used alone or in combination with other herbs or caffeine produces an average weight loss of 0.9 kg/month, ephedra and ephedrine have been widely used as dietary supplements to enable weight loss (17). However, an increasing number of adverse reactions to ephedra have been reported. Dietary supplements containing the ephedrine alkaloid are associated with mortality as a result of adverse reactions, including myocardial infarction, cardiac arrhythmia, hypertension and stroke (18,19). The U.S. Food and Drug Administration (FDA) have banned the use of non-prescribed drugs containing ephedra or ephedra alkaloids, which has lead to difficulties for the use and development of ephedra drugs. To resolve this problem, novel pharmacological effects of ephedra should be investigated. In addition to ephedrine alkaloids, there are other substances in ephedra, such as polysaccharides, organic acids, flavonoids and tannins (20–22). These substances are anti-free radical and may lower blood pressure and sugar to affect fat metabolism (23–25). Therefore, the current study investigated the impact of ephedra extractions, including ephedrine alkaloids, ephedra polysaccharides and ephedra non-alkaloids, on hyperlipidemia and evaluated the safety of the different extractions from *Ephedra sinica* Stapf.

## Materials and methods

### Preparation of extractions from Ephedra

Ephedra samples (batch no. 0909013; Xianning Kangjin Chinese Herbal Medicine Co., Ltd., Xianning, China) were ground into a coarse powder and fully dissolved in 1% sodium hydroxide solution for 30 min, followed by reflux-extraction with dichloromethane. Subsequently, the dichloromethane extracting solution and residue were obtained. The dichloromethane extracting solution was then further concentrated and extracted using an equal volume of 2% hydrochloric acid, followed by separation of acidic aqueous solution and dichloromethane solution. The acidic aqueous solution was adjusted to neutral and concentrated, in order to obtain ephedrine alkaloids by freeze drying. In addition, the dichloromethane fraction was concentrated to obtain the lipophilic non-alkaloid product. The residue was extracted with double distilled water and precipitated with 95% alcohol to obtain ephedra polysaccharide. The recovered alcohol was freeze-dried to obtain ephedra non-alkaloid, which was then merged with the lipophilic non-alkaloid product for later use as ephedra non-alkaloid. Sodium hydroxide, dichloromethane and hydrochloric acid were all purchased from the 3rd Branch of Tianjin Chemical Reagent Co., Ltd (Tianjin, China).

### Experimental animals and design

A total of 48 male Kunming mice, weighing 18–22 g, were purchased from the Wuhan Institute of Biological Products (Wuhan, China). All the animals were acclimatized to laboratory conditions for seven days, during which they were fed a commercial pellet diet and provided with water *ad libitum*. Experiments were conducted under specific pathogen-free conditions. The animal care and use procedures applied in the study were in accordance with the guidelines established by the Animal Ethics Committee of Wuhan Hospital of Traditional Chinese Medicine (Wuhan, China).

The 48 mice were randomly divided into six groups, which included the normal control (G1; n=8), model control (G2; n=8), positive control (G3; n=8), ephedrine alkaloid (G4; n=8), ephedra polysaccharide (G5; n=8) and ephedra non-alkaloid (G6; n=8) groups. Animals in the normal control group were fed a standard basal diet, while the mice in the other five groups were fed a high fat diet (78.8% basal diet, 10% egg yolk, 10% lard, 1% cholesterol, 0.2% bile salt) for three consecutive weeks to establish the hyperlipidemic model. Successful model establishment was confirmed by measurement of the lipid levels.

Mice in the positive control group were administered 6.7 mg/kg simvastatin daily (batch no. 08048; Hangzhou MSD Pharmaceutical Co., Ltd., Hanghzou, China) for four consecutive weeks. Mice in the ephedrine alkaloid, ephedra polysaccharide and non-alkaloid groups were orally administered 1.26 mg/g respective extractions, once per day for four consecutive weeks. Mice in the normal control and model control groups were administered an equal volume of normal saline. The animals were weighed weekly. After four weeks of administration, blood was collected from the eyeballs of mice following fasting for 12 h, and the serum was separated by centrifugation at 2,200 × g for 15 min at 4°C. The mice were euthanized by cervical dislocation, without the use of anesthetic. Organs, including the heart, liver, spleen, lung and kidney, were excised and frozen until required for analysis. Organ coefficients (ratio of organ to body weight) were calculated according to the following formula: Organ coefficient = organ weight (mg)/body weight (g).

### Determination of the serum lipid levels

Serum concentrations of TC, TG and HDL cholesterol (HDL-C) were measured by TC Assay kit, TG Assay kit and HDL Assay kit (Shanghai Mingdian Biological Engineering Co., Ltd., Shanghai, China) respectively, according to the manufacturer’s instructions, using an RT-9600 semi-automatic biochemical analyzer (Shenzhen Leidu Life Science Co., Ltd. Nanning, China).

### Evaluation of antioxidant capacity and liver function

Activity levels of superoxide dismutase (SOD), alanine aminotransferase (ALT) and aspartate aminotransferase (AST), as well as the level of malondialdehyde (MDA) in the serum, were evaluated by SOD Assay kit, ALT Assay kit, AST Assay kit and MDA Assay kit (Nanjing Jiancheng Bioengineering Institute, Nanjing, China) respectively, according to the manufacturer’s instructions.

### Observations of liver morphology

Liver morphologies in the animals from the six groups were observed. The largest lobe of the liver was fixed with neutral formalin, embedded in paraffin, divided into sections (4–5 μm) and stained by routine hematoxylin and eosin (H&E). The morphological changes in the hepatic tissue were observed under a light microscope (BX51; Olympus Corporation, Tokyo, Japan).

### Acute toxicity

A total of 100 male Kunming mice (18–22 g of weight) were used for the acute toxicity study, with 20 mice in each of the ephedra polysaccharide and non-alkaloid groups, and 60 mice in the ephedra alkaloid group (10 from each dose group are shown in Table I). Initially, nine mice were randomly divided into three groups and orally administered ephedrine alkaloids, ephedra polysaccharides and ephedra non-alkaloids, respectively. The mice were monitored to record any clinical signs of toxicity, the time taken to the onset of these symptoms and the time period until mortality. Results from the initial exposure were used to select the subsequent dose, and the up-and-down procedure was used to estimate the lethal dose (26). The selected dosages of ephedra alkaloids were 289, 413, 590, 843, 1,204 and 1,720 mg/kg, the dosage of ephedra polysaccharides was 800 mg/kg and the dosage of ephedra non-alkaloids was 4,168 mg/kg. The mean lethal dose (LD_50_) was calculated using the modified Spearman-Karber method (27). At 0 and 2 h after oral administration, the mice were externally prewarmed for 5 min at 39°C, and the systolic blood pressure (SBP) and heart rate (HR) of the mice were measured using the tail-cuff method (BP98A; Softron Co., Ltd., Tokyo, Japan).

### Statistical analysis

Results are expressed as the mean ± standard deviation, and statistical comparisons were performed using the Student’s t-test. P<0.05 was considered to indicate a statistically significant difference. All statistical tests were performed using SPSS 16.0 software (SPSS., Inc., Chicago, IL, USA).

## Results

### Effects of the extractions on the weight and organ coefficients

**Figure 1:**
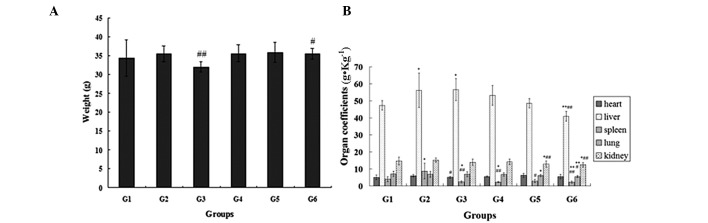
Changes in (A) body weight and (B) organ coefficients in the mice at week four following drug administration in the normal control (G1), model control (G2), positive control (G3), ephedrine alkaloid (G4), ephedra polysaccharide (G5) and ephedra non-alkaloid (G6) groups. ^*^P<0.05 and ^**^P<0.01, vs. normal control group; ^#^P<0.05 and ^##^P<0.01, vs. model control group.

No statistically significant differences were observed in the body weight between the normal control group and the other groups (P>0.05). Compared with the model control group, the weight of the mice in the positive control (P<0.01) and non-alkaloid groups (P<0.05) was significantly lower after four weeks of drug administration (Fig. 1A).

The liver and spleen coefficients in the model control group were significantly higher compared with those in the normal control group (P<0.05). In addition, compared with the model control group, the liver (P<0.01), spleen (P<0.01), lung (P<0.05) and kidney (P<0.01) coefficients were markedly reduced in the ephedra non-alkaloid group. In addition, the spleen (P<0.05) and kidney (P<0.01) coefficients were notably reduced in the ephedra polysaccharide group compared with the model control group. The spleen (P<0.01) coefficient was also significantly lower in the ephedrine alkaloid group when compared with the model control group (Fig. 1B).

### Effects of the extractions on the levels of serum lipids

**Figure 2:**
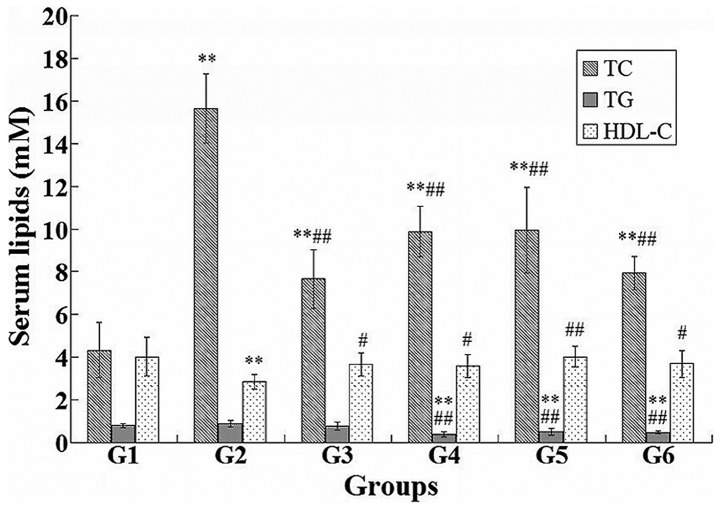
Differences in the serum lipids, TC, TG and HDL-C, among the normal control (G1), model control (G2), positive control (G3), ephedrine alkaloid (G4), ephedra polysaccharide (G5) and ephedra non-alkaloid (G6) groups. ^*^P<0.05 and ^**^P<0.01, vs. normal control group; ^#^P<0.05 and ^##^P<0.01, vs. model control group. TC, total cholesterol; TG, triglycerides; HDL-C, high-density lipoprotein cholesterol.

Statistical analysis of the differences in the levels of serum lipids among the mice in the six groups was performed (Fig. 2). The administration of ephedrine alkaloids, ephedra polysaccharides and non-alkaloids resulted in a significant decrease in the levels of TC (P<0.01) and TG (P<0.01), and an increase in the level of HDL-C (P<0.05), when compared with the model control group. The same changes were observed in the levels of TC and HDL-C in the positive control group. Compared with the normal control group, the level of TC was significantly higher in the other five groups (P<0.01), whereas the level of TG was significantly lower in the ephedrine alkaloid, ephedra polysaccharide and non-alkaloid groups (P<0.01). The level of HDL-C was markedly reduced in the model control group compared with the normal control group (P<0.01).

### Effects of the extractions on antioxidant capacity and liver function

**Figure 3:**
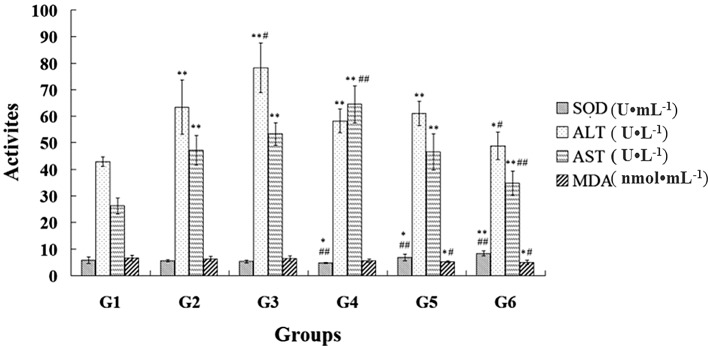
Effects of ephedra extractions on the antioxidant capacity and liver function in the mice from the normal control (G1), model control (G2), positive control (G3), ephedrine alkaloid (G4), ephedra polysaccharide (G5) and ephedra non-alkaloid (G6) groups. ^*^P<0.05 and ^**^P<0.01, vs. normal control group; ^#^P<0.05 and ^##^P<0.01, vs. model control group. SOD, superoxide dismutase; ALT, alanine aminotransferase; AST, aspartate aminotransferase; MDA, malondialdehyde.

Changes in the MDA content, and the activity levels of SOD, ALT and AST among the six groups are shown in Fig. 3. Compared with the model control group, the ephedra polysaccharide and non-alkaloid groups revealed a significantly increased activity of SOD and a reduced content of MDA (P<0.05). Compared with the normal control group, the activity of SOD was significantly enhanced and the content of MDA was decreased in the ephedra polysaccharide and non-alkaloid groups (P<0.05).

Compared with the model control group, the non-alkaloid group demonstrated a decrease in the activity levels of ALT (P<0.05) and AST (P<0.01), whereas the simvastatin (positive control) and ephedrine alkaloids significantly increased the activity levels of ALT (P<0.05) and AST (P<0.01), respectively. The activity levels of ALT and AST were significantly higher in the other five groups compared with those in the normal control group (P<0.01).

### Morphology of the liver and biopsy of the liver tissue

**Figure 4:**
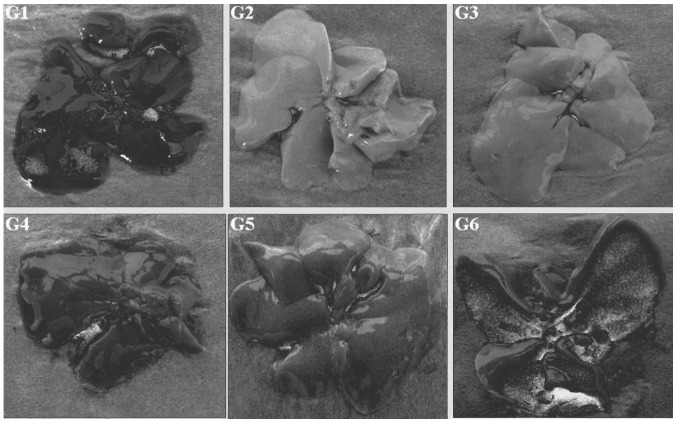
Macro-morphologies of the livers in the normal control (G1), model control (G2), positive control (G3), ephedrine alkaloid (G4), ephedra polysaccharide (G5) and ephedra non-alkaloid (G6) groups.

Macro-morphologies of the livers in the six groups are shown in Fig. 4. The liver in the normal control group was pinkish-brown, soft and elastic with a sharp edge, smooth capsule and cut surface. In the model control group, the livers of the mice showed a varying degree of fat infiltration (liver appearance was cream-colored and greasy with a blunt edge) and hepatomegaly. Compared with the model control group, fatty degeneration of the liver in the other four groups was improved to varying degrees, particularly in the ephedra alkaloid and ephedra non-alkaloid groups.

**Figure 5:**
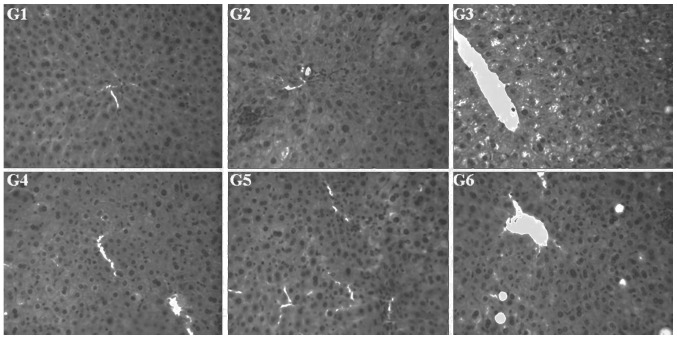
Morphological changes in the hepatic tissues of mice in the normal control (G1), model control (G2), positive control (G3), ephedrine alkaloid (G4), ephedra polysaccharide (G5) and ephedra non-alkaloid (G6) groups following hematoxylin and eosin staining (magnification, ×100).

The H&E staining results (Fig. 5) showed that the structure of the hepatic lobule in the normal control group was clear and there were no significantly degenerated cells, inflammatory cells or lipid droplets present in the tissue. However, in the model control group, the structure of the hepatic lobule was disordered and there was marked swelling of the liver cells, focal necrosis and widespread distribution of lipid droplets. In the other four groups, there was no significant swelling of the liver cells, enlargement of liver cell volume, fatty degeneration or necrosis. Although sections of the lobular structures in the liver cells were unclear, the livers in these four groups revealed less fatty degeneration of the liver cells and smaller lipid droplets.

### Acute toxicity

**Figure 6:**
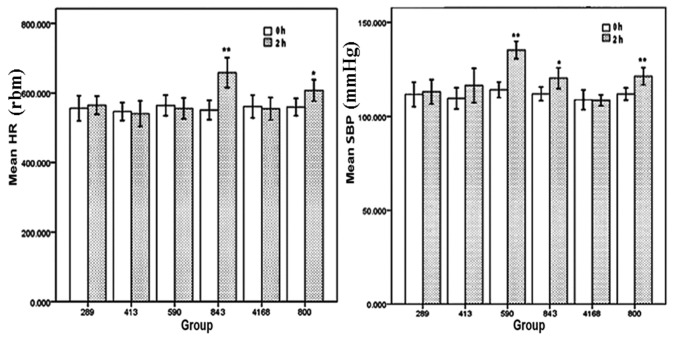
Mean HR and SBP of the mice orally administered 289, 413, 590 or 843 mg/kg ephedra alkaloids, 4,168 mg/kg ephedra non-alkaloids, or 800 mg/kg ephedra polysaccharides at 0 and 2 h after administration. ^*^P<0.05 and ^**^P<0.01, vs. 0 h. HR, heart rate; SBP, systolic blood pressure.

All the mice survived in the 800 mg/kg ephedra polysaccharide group (n=20) and 4,168 mg/kg ephedra non-alkaloid group (n=20), and no abnormal behavior was observed. The acute toxicity of ephedra alkaloids to the mice was assessed by determination of the seven-day LD_50_ value. The calculated LD_50_ was 610 mg/kg with a 95% confidence interval of 499–745 mg/kg (Table I). No abnormal clinical symptoms were observed in the mice administered 289 mg/kg ephedra alkaloids; however, one mouse administered 413 mg/kg ephedra alkaloids died, and all mice exposed to 843 mg/kg ephedra alkaloids showed hyperactive behavior. The HR and SBP of the mice orally administered 289, 413, 590 and 843 mg/kg ephedra alkaloids, 4,168 mg/kg ephedra non-alkaloids, or 800 mg/kg ephedra polysaccharides at 0 and 2 h after administration are shown in Fig. 6. A significant increase in the HR was observed in the 843 mg/kg (P<0.01) ephedra alkaloid and 800 mg/kg (P<0.05) ephedra polysaccharide groups at 2 h following administration. In addition, the SBP of the mice in the 590 mg/kg (P<0.01), 843 mg/kg (P<0.05) ephedra alkaloid and 800 mg/kg ephedra polysaccharide groups (P<0.01) were significantly increased at 2 h following administration.

## Discussion

Currently, there are various hypolipidemic drugs available, including statins, fibrates and bile acid sequestrants; however, they all exhibit numerous side effects. Therefore, there is an urgent requirement for the development of hypolipidemic drugs from natural resources. In the present study, ephedra extractions were demonstrated to improve lipid metabolism in a diet-induced hyperlipidemia mouse model.

Compared with the model control group, ephedra extractions significantly reduced the levels of TC and TG, and increased the level of HDL-C in the serum. Compared with the positive control group, ephedra polysaccharides and non-alkaloids had the advantage of lowering the TG level. A low level of HDL-C has been documented as an indicator of high risk for cardiovascular disease, and an increase in the level of HDL-C may potentially contribute to anti-atherogenicity, inhibit LDL oxidation and protect endothelial cells from the cytotoxic effects of OxLDL (28,29). Therefore, the present results indicate that ephedra non-alkaloids may protect against cardiovascular disease by increasing the level of HDL-C.

The livers in the model control group revealed a varying degree of fat infiltration and hepatomegaly; however, this condition was improved in the other four groups, particularly the ephedra alkaloid and non-alkaloid groups. The liver coefficient of the non-alkaloid group was markedly lower compared with the other groups, which indicated that the hepatomegaly had improved significantly. The lipid droplets were smaller and less intensive in the ephedrine alkaloid, ephedra polysaccharide and non-alkaloid groups, which suggested that ephedra extractions lowered the lipid levels. Ephedra polysaccharides and ephedra non-alkaloids may scavenge lipids and reduce the absorption of fat to decrease the levels of lipids.

In hypercholesteremia, the activity of SOD is decreased and the levels of free radicals and MDA are increased. Polyphenols and flavonoids may scavenge free radicals, including hydroxyl and superoxide anions, to inhibit lipid peroxidation and improve lipid profiles (30–33). These drugs are also known to stimulate catalase and SOD gene transcription, and decrease the MDA concentration (34,35). In the current study, ephedra polysaccharides and non-alkaloids significantly increased the activity of SOD and decreased the level of MDA, which indicated that ephedra polysaccharides and non-alkaloids were able to remove free radicals. Lipid peroxidation, in the form of increased MDA production, has been observed in previous studies, and serum levels of MDA have been shown to correlate with the severity of chronic hepatitis, indicating increased oxidative stress in patients with nonalcoholic steatohepatitis (36,37). Not only the cell membrane lipids, but also the cell membrane proteins, can be oxidized by free radicals (38), which are responsible for the damage of hepatic cell structure and function and lipid metabolism disorder in the liver. SOD is the first line of defense against oxygen-derived free radicals, converting superoxide anions into H_2_O_2_ and reducing the destruction of hydrogen and lipid hydroperoxides (39). The results of the present study revealed that ephedra polysaccharides and ephedra non-alkaloids are able to remove free radicals to protect the liver. In the ephedra polysaccharide and non-alkaloid groups, there were no significantly swollen liver cells and fatty degeneration of the hepatic cells. In addition, the lobular structure of the liver cells was clearer compared with that of the model control and positive control groups. Therefore, ephedra non-alkaloids may exhibit protective effects on the liver.

The liver plays a critical role in the normal metabolism of energy substrates, particularly lipid metabolism. AST and ALT are the main indicators for evaluating liver function and the response to liver injury (40). Compared with the normal and model control groups, simvastatin and ephedrine alkaloids significantly increased the activity levels of ALT and AST (P<0.05), indicating that these compounds had side effects on the liver. However, ephedra non-alkaloids were shown to decrease the activity levels of ALT (P<0.05) and AST (P<0.01), which indicated that ephedra non-alkaloids may restore liver function.

Since the administration of ephedra non-alkaloids is limited by the solubility and dose volume, the LD_50_ was unable to be measured, indicating that the toxicity of ephedra non-alkaloids is extremely low. In the acute toxicity study, the maximum tolerated dose of oral ephedra non-alkaloids in the mice was 367.5-fold larger than the maximal dosage in humans, as specified in the Pharmacopoeia of the People’s Republic of China (41) (9 g crude drug). In addition, the SBP and HR of the mice did not notably change and no abnormal behavior was observed. However, the SBP and HR of the mice administered high dosages of ephedra polysaccharides and alkaloids were significantly increased. These results demonstrated that ephedra non-alkaloids may not induce cardiovascular side effects and are safe for use.

In conclusion, ephedra non-alkaloids are relatively safe and have the potential to improve hyperlipidemia. The protective effects of ephedra non-alkaloids may be due to the prevention of free radical generation, as well as recuperation of liver function during liver damage.

## Figures and Tables

**Table I:** Mortality rate of the mice treated with graded doses of ephedra alkaloids.

Dose (mg/kg)	Route	Animals (n)	Mortality (n)
289	Orally	10	0
413	Orally	10	3
590	Orally	10	6
843	Orally	10	7
1,204	Orally	10	8
1,720	Orally	10	10

## References

[1] Lind L, Lithell H (1993). Decreased peripheral blood flow in the pathogenesis of the metabolic syndrome comprising hypertension, hyperlipidemia, and hyperinsulinemia. Am Heart J.

[2] Colomé C, Martínez-González J, Vidal F, de Castellarnau C, Badimon L (2000). Small oxidative changes in atherogenic LDL concentrations irreversibly regulate adhesiveness of human endothelial cells: effect of the lazaroid U74500A. Atherosclerosis.

[3] Steinberg D (1997). Lewis A. Conner Memorial Lecture. Oxidative modification of LDL and atherogenesis. Circulation.

[4] Cockerill GW, Saklatvala J, Ridley SH (1999). High-density lipoproteins differentially modulate cytokine-induced expression of E-selectin and cyclooxygenase-2. Arterioscler Thromb Vasc Biol.

[5] Khan BV, Harrison DG, Olbrych MT, Alexander RW, Medford RM (1996). Nitric oxide regulates vascular cell adhesion molecule 1 gene expression and redox-sensitive transcriptional events in human vascular endothelial cells. Proc Natl Acad Sci USA.

[6] Assmann G, Schulte H (1988). The Prospective Cardiovascular Münster (PROCAM) study: prevalence of hyperlipidemia in persons with hypertension and/or diabetes mellitus and the relationship to coronary heart disease. Am Heart J.

[7] Chiu JH, Abdelhadi RH, Chung MK (2005). Effect of statin therapy on risk of ventricular arrhythmia among patients with coronary artery disease and an implantable cardioverter-defibrillator. Am J Cardiol.

[8] Maron DJ, Fazio S, Linton MF (2000). Current perspectives on statins. Circulation.

[9] Kohli P, Desai NR, Giugliano RP (2012). Design and rationale of the LAPLACE-TIMI 57 trial: a phase II, double-blind, placebo-controlled study of the efficacy and tolerability of a monoclonal antibody inhibitor of PCSK9 in subjects with hypercholesterolemia on background statin therapy. Clin Cardiol.

[10] Armitage J (2007). The safety of statins in clinical practice. Lancet.

[11] Neuvonen PJ, Niemi M, Backman JT (2006). Drug interactions with lipid-lowering drugs: mechanisms and clinical relevance. Clin Pharmacol Ther.

[12] Conforti A, Magro L, Moretti U (2006). Fluvastatin and hepatic reactions: a signal from spontaneous reporting in Italy. Drug Saf.

[13] Abourashed EA, El-Alfy AT, Khan IA, Walker L (2003). Ephedra in perspective - a current review. Phytother Res.

[14] Soni MG, Carabin IG, Griffiths JC, Burdock GA (2004). Safety of ephedra: lessons learned. Toxicol Lett.

[15] Kuang H, Yonggang X, Yang B, Wang Q, Wang Y (2011). Screening and comparison of the immunosuppressive activities of polysaccharides from the stems of Ephedra sinica Stapf. Carbohydrate Polymers.

[16] Xia Y, Kuang H, Yang B, Wang Q (2011). Optimum extraction of acidic polysaccharides from the stems of Ephedra sinica Stapf by Box-Behnken statistical design and its anti-complement activity. Carbohydrate Polymers.

[17] Shekelle PG, Hardy ML, Morton SC (2003). Efficacy and safety of ephedra and ephedrine for weight loss and athletic performance: a meta-analysis. JAMA.

[18] Kalman D, Incledon T, Gaunaurd I, Schwartz H, Krieger D (2002). An acute clinical trial evaluating the cardiovascular effects of an herbal ephedra-caffeine weight loss product in healthy overweight adults. Int J Obes Relat Metab Disord.

[19] Haller CA, Benowitz NL (2000). Adverse cardiovascular and central nervous system events associated with dietary supplements containing ephedra alkaloids. N Engl J Med.

[20] al-Khalil S, Alkofahi A, el-Eisawi D, al-Shibib A (1998). Transtorine, a new quinoline alkaloid from Ephedra transitoria. J Nat Prod.

[21] Purev O, Pospísil F, Motl O (1988). FPaOM: Flavonoids from Ephedra sinica Stapf. Collect Czech Chem Commun.

[22] Starratt AN, Caveney S (1996). Quinoline-2-carboxylic acids from Ephedra species. Phytochemistry.

[23] Konno C, Mizuno T, Hikino H (1985). Isolation and hypoglycemic activity of ephedrans A, B, C, D and E, glycans of Ephedra distachya herbs. Planta Med.

[24] Chumbalov TK, Chekmeneva LN, Polyakov VV (1977). Phenolic acids of Ephedra equisetina. Chemistry of Natural Compounds.

[25] Zhang L, Zou G, Yang T (2000). Studies on extraction of water-soluble polysaccharides and the function of cleaning oxygen free-radical function of ephedra. Amino Acids and Biotic Resources.

[26] OECD (Organisation for Economic Co-operation and Development) (2001). OECD Guidelines for the Testing of Chemicals. Guideline 425: Acute Oral Toxicity-Up-and-Down Procedure.

[27] Karber G (1931). Determination of LD50. Arch Exp Pathol Pharma.

[28] Wilson PW, Abbott RD, Castelli WP (1988). High density lipoprotein cholesterol and mortality. The Framingham Heart Study Arteriosclerosis.

[29] Assmann G, Nofer JR (2003). Atheroprotective effects of high-density lipoproteins. Annu Rev Med.

[30] Rice-Evans CA, Miller NJ, Bolwell PG, Bramley PM, Pridham JB (1995). The relative antioxidant activities of plant-derived polyphenolic flavonoids. Free Radic Res.

[31] Tripathi YB, Singh BK, Pandey RS, Kumar M (2005). BHUx: a patent polyherbal formulation to prevent atherosclerosis. Evid Based Complement Alternat Med.

[32] Ljubuncic P, Dakwar S, Portnaya I (2006). Aqueous extracts of Teucrium polium possess remarkable antioxidant activity in vitro. Evid Based Complement Alternat Med.

[33] Punitha IS, Rajendran K, Shirwaikar A, Shirwaikar A (2005). Alcoholic stem extract of Coscinium fenestratum regulates carbohydrate metabolism and improves antioxidant status in streptozotocin-nicotinamide induced diabetic rats. Evid Based Complement Alternat Med.

[34] Toyokuni S, Tanaka T, Kawaguchi W (2003). Effects of the phenolic contents of Mauritian endemic plant extracts on promoter activities of antioxidant enzymes. Free Radic Res.

[35] Ralay Ranaivo H, Rakotoarison O, Tesse A (2004). Cedrelopsis grevei induced hypotension and improved endothelial vasodilatation through an increase of Cu/Zn SOD protein expression. Am J Physiol Heart Circ Physiol.

[36] Paradis V, Mathurin P, Kollinger M (1997). In situ detection of lipid peroxidation in chronic hepatitis C: correlation with pathological features. J Clin Pathol.

[37] Yadav D, Hertan HI, Schweitzer P, Norkus EP, Pitchumoni CS (2002). Serum and liver micronutrient antioxidants and serum oxidative stress in patients with chronic hepatitis C. Am J Gastroenterol.

[38] Kako KJ (1987). Free radical effects on membrane protein in myocardial ischemia/reperfusion injury. J Mol Cell Cardiol.

[39] Harris ED (1992). Regulation of antioxidant enzymes. FASEB J.

[40] Limdi JK, Hyde GM (2003). Evaluation of abnormal liver function tests. Postgrad Med J.

[41] State Pharmacopoeia Commission of the People’s Republic of China (2005). Pharmacopoeia of the People’s Republic of China.

